# Simultaneous Pattern Visual Evoked Potential and Pattern Electroretinogram in Strabismic and Anisometropic Amblyopia

**Published:** 2011-01-01

**Authors:** J Heravian, R Daneshvar, F Dashti, A Azimi, H Ostadi Moghaddam, A A Yekta, H Esmaily

**Affiliations:** 1 Department of Optometry, School of Paramedical Sciences, Mashhad, Iran; 2 Eye Research Center, Khatam Anbia Hospital, Mashhad, Iran; 3 Department of Social Medicine, School of Health, Mashhad University of Medical Sciences, Mashhad, Iran

**Keywords:** Strabismic amblyopia, Anisometropic amblyopia, PVEP, PERG

## Abstract

**Background:**

Amblyopia is a relatively common condition in which visual acuity through an eye is subnormal despite no overt pathology. Pattern visual evoked potential (PVEP) can detect any defect from optic nerve to occipital cortex and pattern electroretinogram (PERG) can detect retinal defects specially the ganglion layers. This study was performed to evaluate the cortical and retinal activity in strabismic and anisometropic amblyopia.

**Methods:**

PVEP and PERG were recorded simultaneously in 40 amblyopes (20 strabismics and 20 anisometropics) and 20 normal control subjects. Normal subjects were age and sex matched with patients.

**Results:**

The P_100_ latency in PVEP was increased in both groups of patients but the P_100_ amplitude was reduced only in anisometropic group. In PERG, the amplitude of P_50_ was reduced in all patients with no significant change in latency.

**Conclusion:**

Beside reduced PVEP responses in strabismic and anisometropic amblyopia, the activity of retina reduced too. It is likely that retinal impulses can affect the development of visual system.

## Introduction

Amblyopia is an acquired unilateral or bilateral decrease of visual acuity for which no obvious structural or pathologic cause can be detected by physical examination of the eye. The most common types of amblyopia are strabismic and anisometropic amblyopia. The purpose of electrophysiologic studies in amblyopia is an understanding of the mechanism of decreased visual acuity and revealing the location of major defects and their depth. In many studies, pattern visual evoked potential (PVEP) recorded from human amblyopic eyes showed attenuated amplitudes and prolonged latencies.[1][2][3] Although the loss of visual acuity in amblyopia is considered to be cortical in origin,[4][5] it remains unclear whether the retina is also affected in humans with amblyopia or not.

Feng et al using multifocal pattern visual evoked potential ((MFVEP) and multifocal pattern electroretinogram (MFERG) in 42 amblyopes showed attenuated amplitude of both MFERG and VEP in amblyopic eyes with the prolonged latency of MFVEP but no change in MFERG latency.[6] Arden et al. argued that the reduction of pattern electroretinogram (PERG) in amblyopia occurs without a corresponding reduction in focal electroretinogram (ERG) and this reduction may differ according to the type of amblyopia.[7] On the other hand Guttob and his coworkers[8] and Hess et al.[9] stated that PERG is normal in any type of amblyopia. This study was undertaken to investigate the effect of amblyopia on both the retinal and cortical pattern responses.

## Material and Methods

The patients for this study comprised 40 amblyopes (20 strabismic and 20 anisometropic). Amblyopia was defined as at least 2 lines differences in best corrected Snellen acuity between eyes or an absolute best corrected Snellen visual acuity of 20/30 or less, in the absence of obvious structural pathology. The 20 subjects who comprised our normal control group were age and sex matched with the patients and were selected to have no ophthalmologically detected pathology with normal ocular history and normal visual acuity. The study protocol was approved by the Ethical Committee of Mashhad University of Medical Sciences and adhered to tenets of Helsinki Declaration. The procedure was explained to all subjects and informed consent was obtained before examination. Subjective visual acuity was measured monocularly and binocularly by a standard Snellen distance chart (Clement Clark International, UK). The subjects were refracted to ensure an exact optical correction. The orthoptic evaluation included objective measuring of the angle of deviation and determination of fixation characteristics was established using visoscopy. Stereoacuity was measured with the TNO Stereotest (Clement Clark International, UK) for all subjects. Suppression was checked with the worth 4-dot test. Biomicroscopic and ophthalmoscopic investigation showed clear media and no fundus abnormalities. All patients and subjects had stable fixation.

The PVEP recording equipment consisted of a Roland Reti (Model ISXEV 60, Germany) signal averager connected to a 2 to 8 channels amplifier for storing and summating the waves. The stimulus was a black and white check size of 48 min arc at a viewing distance of one meter. The mean screen luminance was 100 cd/m2 with a contrast of 99% and full field display. The temporal frequency was 1.5 Hz (3 reversals per second). The mean luminance of the test room was 80 cd/m^2^ and recording condition was in accordance with International Society of Clinical Electrophysiology of Vision (ISCEV) standards. The amplifier band-pass filters were set at 1-50Hz. In recording the PVEP, the active electrode was positioned one inch above the inion (o_z_). Referencing was to the centre of the forehead with a ground electrode on the vertex of the head (c_z_). In recording the PERG, the active electrode (DTL fiber) was positioned on the lower fornix in contact with the limbus, referenced to the outer cantus of the ipsilateral eye with a ground electrode on the forehead. The inter electrode impedance was maintained below 5 K Ohm in all recordings. The PVEP was recorded first binocularly and then monocular PVEPs and PERGs were recorded simultaneously for each subject. In each recording, 200 sweeps were averaged. All electrophysiological tests were performed at Electrophysiology Laboratory of Khatam Anbia Eye Hospital, a tertiary eye hospital in northeast of Iran. All tests were performed with the subjects/patients wearing the best refractive correction. Finally the data were analyzed with SPSS Version 11.5 Inc, Chicago, IL, USA. A p value of < .05 was considered significant. The data analysis was performed, using Pearson correlation coefficient, Independent t-test, One- way (ANOVA), Dunnet and Tukey tests.

## Results

Table 1 shows the mean and standard deviation of the P_100_ latency and amplitude of the amblyopic and non-amblyopic eyes and binocular responses in both strabismic and anisometropic groups. Table 2 shows the normal subjects data for the P_100_ amplitudes and latencies. There was a significant difference for the P_100_ latency between both group of amblyopes and normal subjects (p=0.001 for the anisometropic group and p<0.001 for the strabismic group). However, there was no significant difference for the P_100_ amplitude between both group of amblyopes and normal subjects (p=0.203 for the anisometropic group and p=0.935 for the strabismic group).

**Table 1:** Mean and standard deviation of P_100_ latency and amplitude for the amblyopic and nonamblyopic eyes and binocular responses in strabismic and anisometropic groups

** Eye**	**Strabismic amblyopia**		**Anisometropic amblyopia**	
	**P_100_latency(ms)**	**P_100_mplitude (µv)**	**P_100_atency** **(ms)**	**P_100_amplitude(µv)**
Amblyopic eye	115.5±10.2	17.1±25.1	109.6±9.5	10.8±6.8
Non-amblyopic eye	107.5±7.9	15.1±5.6	104.4±7.3	12.1±0.5
Binocular responses	109±7.448	18.95±7.028	105.2±6.504	13.53±6.739

**Table 2:** Mean and standard deviation of P_100_ latency and amplitude in monocular and binocular responses in normal subjects

**Eye**	**P_100_ latency (ms)**	**P_100_ amplitude (μv)**
Dominant eyes responses	99.9±3.2	18.6±7.1
Binocular responses	94.4±19.1	15.91±4.3

Independent t-test was performed for amplitudes and latencies between the two groups of amblyopes. There was no significant difference between the response of the amblyopic eyes of the two groups of patients (p=0.268) for the P_100_ amplitude and p=0.641 for the P_100_ latency of the two groups of patients).

There was no significant difference between the P_100_ amplitude of the binocular response of the anisometrops and normal subjects (p=0.446) but the difference was significant for the P_100_ latency (p=0.026). There was also no significant difference between the P_100_ amplitude of the binocular response of the strabismic amblyopes and normal subjects (p=0.274), but there was significant difference for the P_100_ latency (p=0.002) between strabismic amblyopes and normal subjects. There was a significant difference in binocular P_100_ amplitude between the two groups of amblyopes (p=0.020), with no significant difference for the latencies (p=0.626).

Table 3 shows the mean and standard deviation of the P_50_ amplitudes and latencies in the strabismic and anisometropic groups. There was a significant difference between P_50_ amplitude for the amblyopic eye of strabismic and normal subjects (p<0.001) with no significant difference for P_50_ latencies (p=0.460). In addition, there was a statistically significant difference between P_50_ amplitude for the amblyopic eye of anisometrops and normal subjects (p<0.001) but no significant difference between P_50_ latencies (p=0.871). There was no significant difference between P_50_ amplitude (p=0.719) and P_50_ latency (p=0.215) for the 2 groups of patients. Table 4 shows the normal subjects data for the P_50_ amplitudes and latencies.

**Table 3:** Mean and standard deviation of P_50_ amplitude and latency in amblyopic and non-amblyopic eyes of strabismics and anisometropics groups

**Eye**	**Strabismics group**		**Anisometropics group**	
	**P_50_ latency (ms)**	**P_50_ amplitude (µv)**	**P_50_ latency (ms)**	**P_50_ amplitude (µv)**
Amblyopic eye	52.6±4.4	1.7±0.96	50.4±4.9	1.9±1.13
Non-amblyopic eye	52.6±4.9	3.1±1.3	49.4±5.09	3.5±1.6

**Table 4:** Mean and standard deviation of P_50_ amplitude and latency in normal subjects

**Eye**	**P_50_ latency (ms)**	**P_50_ amplitude (µv)**
Monocular responses	51±2.4	3.7±1.01

P_50_ of PERG, showing a significant correlation (Pearson correlation=0.58; p<0.001).

## Discussion

The results of the PVEP in anisometropic amblyopia suggest that the mean amplitude of P_100_ reduced in comparison with normal subjects and the mean latency was prolonged. These findings confirm previous reports.[1][2][3][10][11] Our findings for the strabismic group, which there was a significant prolonged latency in the amblyopic eyes agree with other studies. A large number of studies using conventional pattern VEP showed both increased latency and reduced amplitude of the responses from the amblyopic eye. These changes are attributed to the cortical patophysiology. One possibility is that the changes of latency and amplitude are caused by an enhancement of magnocellular to paravocellular responses in strabismic amblyopia. Levi showed that the reduction of P_100_ amplitude can be due to reduced cortical neurons which are stimulated by the amblyopic eye.[12] Katz et al. showed that the major effect of strabismus may be to alter the balance of excitory and inhibitory connections to a neuron and it is believed that strabismus selectively reduces local and long-range excitatory connections and lead to reduction in response of cortical cells.[13]

Demer et al. reported that the reduction of amplitude and prolonged latency response of an amblyopic eye is due to inhibitory impulses of the normal eye.[14] In this study, we did not find any significant difference between P_100_ latency and amplitude in two groups of amblyopes which is in agreement with other studies. McKee et al. have emphasized the similarities between the pattern of visual losses in anisometropic and strabismic amblyopia.[15] This promotes the view that irrespective of cause, amblyopia is a condition that varies more in severity than in kind.[16][17] These results indirectly challenge the notion that anisometropic and strabismic amblyopia are distinct neural anomalies with separate etiologies (chronic unilateral blur versus chronic unilateral suppression).[16]

Our results indicate that the binocular PVEP responses of all patients are reduced in comparison with normal subjects. This confirms the findings of other studies.[18][19][20] The study of macaques’ visual system revealed that the primary visual cortex of both strabismic and anisometropic amblyopes had lower binocular neurons.[21][22] Nevertheless, it has been discussed that abnormal binocular interaction occurs in amblyopia in visual cortex and this means that there are binocular cells in visual cortex but they produce smaller responses.[23][24] However, recent physiological investigations seems to support abnormal binocular interactions in visual cortex of amblyopes as opposed to loss of cortical binocularity that had previously been thought to be the case.

We did not find any change in PERG latencies between both groups of amblyopes and normal subjects. This finding supports the results of previous studies.[25][26] Also, we did not find any significant difference on P_50_ latencies between two eyes of patients. This finding confirms that the latencies of PERG responses are mostly constant and rarely change in different diseases of retina and optic nerve.

Tepping et al. in their simultaneous study of PVEP and PERG in amblyopic eyes reported no significant delay in peak latencies in PERG, whereas in the PVEP, latencies were significantly prolonged. They confirmed that a conduction delay of visual information appears unlikely to occur on a retinal level and the total latency delay in the VEP of amblyopic eye is caused by a prolongation of retin-cortical time.[27]

Our results showed that the amplitude of P_50_ was reduced in all patients which agree with other studies. [28][29][30] Manny et al. showed that reduced P_50_ amplitude could be due to reduced function of retinal ganglion cells.[28] It is believed that X ganglion cells are the major origin of generating PERG responses.

Armington et al. reported that the reduced PERG responses in amblyopia is indicative of malfunction of photopic mechanism of amblyopic eye. It is believed that process of contrast information in human visual system begins from retinal ganglion cells.[31] Jacobson et al showed that the X ganglion cells need optimal optical focus to develop properly as cortical neurons need.[32] We also found that the P_50_ amplitude increases parallel to the visual acuity.

The study of mice PERG suggests that although retinal ganglion cell axons activity plays a role in PERG generation, a Muller cell component can not be excluded, since Muller cells can passively generate electric currents in response to extracellular modulation of K^+^ ions produced by active retinal neurons.[33]

Biological studies have shown evidences that in amblyopic eye, the function of neurotransmitters changes and the reduction of P_50_ amplitude can be due to this dysfunction.[25] It has been reported that retinal neurotransmitters have a major role in generating retinal responses. Glutamate is the transmitter released by rods and cones and some bipolar cells. Glutamate acts on ganglion cells to increase the visually evoked responses of both on- and off-sustained ganglion cells but not of transient cells. On the other hand, Acetylcholine enhances the firing of on- and off-transient ganglion cells. Acetylcholine is released by amacrine cells.

The present study showed that there was a significant correlation between P_50_ amplitude of PERG and P_100_ latency of PVEP and as the P_50_ amplitude increased, the P_100_ latency decreased which is in agreement with other studies.[34][35] This result brings the hypothesis that reduced visual cortex function is not only due to reduced cortical cells but also due to reduced retinal responses of the amblyopic eyes.

Yin et al. reported that beside prolonged VEP latencies, the amplitude of PERG reduced in isotropic amblyopic cats. They believed that retinal defect is associated with cortical defects although the retinal defect has a smaller depth.[34] Porciatty et al. reported that the PERG acuity in mice develops postnatally[36]in parallel with visual acuity determined with VEPs.[37] These studies reveal that the defect of retina can eventuate to the malfunction of visual cortex. Tsutsui and coworkers also reported that the reduced amplitude of PVEP can be due to reduction of electrical activity from retina and sites of transmission along the optic pathway[38]

In mammalian visual system, the information of eye specific layers at the thalamic level depends on retinal waves of spontaneous activity,[39] which rely on nicotinic acetylcholine receptor activation.

Porciatti in his studyon mouse PERG has indicated that cholinergic mediated activity in the developing retina is not required for the normal postnatal development of ganglion cells, but is necessary for the anatomical and functional development of the post retinal visual system.[33][40]

The results of this study showed that beside reduced PVEP responses in strabismic and anisometropic amblyopia, the activity of retina is also reduced. It is probable that retinal impulses can affect visual system development.
